# Advances and potential pitfalls of oncolytic viruses expressing immunomodulatory transgene therapy for malignant gliomas

**DOI:** 10.1038/s41419-020-2696-5

**Published:** 2020-06-25

**Authors:** Qing Zhang, Fusheng Liu

**Affiliations:** 1 Brain Tumor Research Center, Beijing Neurosurgical Institute, Capital Medical University, Beijing, 100070 China; 2 Department of Neurosurgery, Beijing Tiantan Hospital Affiliated to Capital Medical University, Beijing, 100070 China; 3 Beijing Laboratory of Biomedical Materials, Beijing, 100070 China

**Keywords:** Cancer immunotherapy, CNS cancer

## Abstract

Glioblastoma (GBM) is an immunosuppressive, lethal brain tumor. Despite advances in molecular understanding and therapies, the clinical benefits have remained limited, and the life expectancy of patients with GBM has only been extended to ~15 months. Currently, genetically modified oncolytic viruses (OV) that express immunomodulatory transgenes constitute a research hot spot in the field of glioma treatment. An oncolytic virus is designed to selectively target, infect, and replicate in tumor cells while sparing normal tissues. Moreover, many studies have shown therapeutic advantages, and recent clinical trials have demonstrated the safety and efficacy of their usage. However, the therapeutic efficacy of oncolytic viruses alone is limited, while oncolytic viruses expressing immunomodulatory transgenes are more potent inducers of immunity and enhance immune cell-mediated antitumor immune responses in GBM. An increasing number of basic studies on oncolytic viruses encoding immunomodulatory transgene therapy for malignant gliomas have yielded beneficial outcomes. Oncolytic viruses that are armed with immunomodulatory transgenes remain promising as a therapy against malignant gliomas and will undoubtedly provide new insights into possible clinical uses or strategies. In this review, we summarize the research advances related to oncolytic viruses that express immunomodulatory transgenes, as well as potential treatment pitfalls in patients with malignant gliomas.

## Facts

1. Oncolytic virus-encoded immunomodulatory transgene therapy for gliomas has yielded beneficial outcomes.
2. Oncolytic virus and tumor-targeting immune modulatory therapies have shown synergistic inhibition of malignant gliomas.
3. Oncolytic virus immunotherapy of malignant gliomas has been used in clinical trials.
4. The combination of stem cell carriers with oncolytic virus therapy for gliomas enhances antitumor efficacy.

## Open questions

1. Can the immune system attack and engulf exogenous viruses?
2. Are glioma stem cells resistant to viral therapy?
3. Can the presence of nontumor cells such as tumor stroma cells impede the spread of oncolytic viruses?
4. In personalized medicine, should potential challenges be considered for the treatment of patients with malignant gliomas?

## Introduction

Glioblastoma (GBM) is both the most aggressive and lethal malignant brain tumor in adults and accounts for more than 30% of intracranial tumors^1,2^. Current standard treatment options for malignant gliomas are multimodal and include surgical resection, postoperative radiotherapy, and concomitant chemotherapy with temozolomide^1,3,4^. Due to the invasive growth and recurrence features of malignant gliomas, the prognosis for patients with malignant gliomas remains extremely poor, with a median survival of nearly 15 months for newly diagnosed patients^5,6^. Thus, more specific, safe, and efficient treatment strategies are required. A growing body of preclinical and clinical data suggest that genetically engineered oncolytic viruses may be effective therapeutic agents used in the treatment of malignant gliomas.

Oncolytic virus (OV) therapy is a novel and promising therapeutic approach for tumors that involves selectively infecting and killing tumor cells. Prior research studies demonstrated that αvβ3 integrin and nectin-1 are required for efficient infection of cells by herpesvirus and adenovirus, respectively^7,8^, and the underlying mechanism may involve OV-induced cell destruction by cancer-specific genetic alteration, as well as sequential virus release and viral infection. It was first reported that tumors could be inhibited or shrunk in patients with cervical cancer and rabies virus positivity^9^. Researchers found that a conditionally and genetically modified replication-competent oncolytic virus was selectively toxic to tumor cells and nontoxic to normal cells^10–12^. Cassel et al. reported that a genetically engineered oncolytic virus was evaluated as an adjunctive therapeutic agent for patients with malignant melanoma^13^. Subsequently, oncolytic viruses, including G207 and HSV1716, were used for clinical research in patients with malignant gliomas in the USA and the UK^14^. In 2015, the FDA approved the use of an oncolytic virus in the USA to treat patients with melanoma^15^. In addition, recent advances in viral therapy, of which the most promising are perhaps viruses such as DNX-2401, PVS-RIPO, and Toca 511, have shown complete durable responses in ~20% of GBM patients who received virus intratumorally^16–18^. However, the clinical trial did not meet its primary endpoints. These encouraging results obtained with PVS-RIPO, Toca 511, and DNX-2401 have been granted a fast track designation by the FDA for expedited drug review processes.

Our research team previously found that the Endo–Angio fusion gene that is expressed in glioma stem cells (GSCs) could be administered via infection by oncolytic HSV-1, which carries an exogenous Endo–Angio fusion gene^19^. Furthermore, Friedman et al. summarized the milestones of oncolytic viruses carrying exogenous genes for cancer treatment^20^. In 2014, we also demonstrated that in animal models of human GSCs, an oncolytic HSV-1 that encodes an endostatin–angiostatin fusion gene could greatly enhance antitumor efficacy compared to HSV-1 without the fusion gene by generating antitumor angiogenic activity^21^. Later, our research team found that viruses that express the suicide gene cytosine deaminase (CD) could significantly enhance antitumor efficacy and prolong the life expectancy of tumor-bearing animals by the subsequent conversion of nontoxic prodrugs into toxic prodrugs^22,23^. Our research team independently developed a novel oHSV-1 containing the CD therapeutic gene. Moreover, a clinical trial using an engineered oHSV-1 (ON-01) injected intratumorally into patients with recurrent or refractory intracranial malignant gliomas is currently ongoing at Beijing Tiantan Hospital (China Clinical Trials registration number: ChiCTR1900022570).

A preclinical study demonstrated that intratumoral administration of OVs could drive the development of systemic antitumor immunity and result in elimination of contralateral tumors^24^. Moreover, oncolytic virus-mediated destruction of the tumor tissues was closely related to robust stimulation of innate antiviral immune responses and adaptive antitumor T-cell responses^25^. To further augment the antitumor effects, certain research studies have transduced immunomodulatory therapeutic transgenes, such as IL-15^26–28^, IL-12^29–32^, IL-4^33,34^, and TRAIL^35,36^, via oncolytic virus, which can effectively inhibit or kill tumor cells through the antitumor immune response. Therefore, oncolytic viruses that encode immunomodulatory transgenes are gradually becoming a research hot spot in the field of glioma treatment. The gene required for oncolytic virus growth is typically placed under the control of a tumor-specific promoter or enhancer. In 2019, Yan et al. constructed a novel recombinant oncolytic adenovirus that expresses IL-15 and under the control of the E2F-1 promoter; it was found that this novel adenovirus could selectively kill tumor cells and exhibit increased antitumor effects both in vitro and in vivo^26^. Moreover, oncolytic viruses that were armed with immunomodulatory transgenes have been evaluated in several preclinical and clinical trials for the treatment of malignant gliomas. Genetically engineered virotherapy for malignant gliomas has so far been proven safe and effective. Nevertheless, many innovative strategies are currently under development to improve intratumoral viral expansion and antitumor efficacy without compromising security. Overall, previous studies on genetically engineered virotherapy with immunomodulatory transgenes have resulted in breakthroughs regarding several difficulties, which may contribute toward new insights into future therapeutics for patients with malignant gliomas.

## Research advancements

Oncolytic viruses are a distinct class of antitumor agents with a unique mechanism of action that selectively replicate and target tumor cells, including GBM cells, affecting the tumor while sparing normal tissues and generating beneficial outcomes in GBM patients. However, diverse factors restrict the effect of virus treatment alone. In recent years, previous studies have inserted immunomodulatory genes into the viral genome, enabling the virus to release immune factors and simultaneously kill tumor cells in a direct manner; further, the antitumor immunologic reaction has been optimized, which has become a new approach for antitumor therapy. Oncolytic viruses that express immunomodulatory transgenes have been evaluated in terms of efficacy and safety in several preclinical and clinical trials for the treatment of malignant gliomas. However, their therapeutic utility in GBM appears to be limited due to several challenges.

**Fig. 1:**
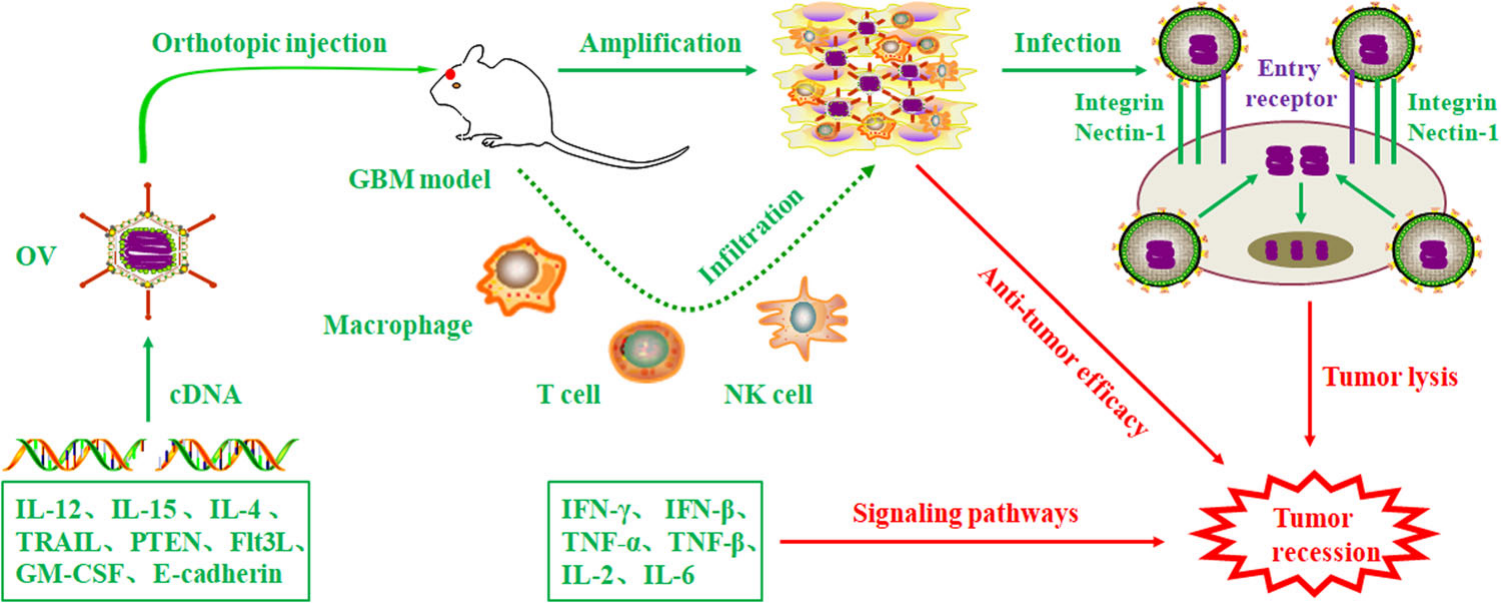
Studies of oncolytic viruses expressing immunomodulatory transgene for GBM treatment. Oncolytic viruses can be transduced to deliver antitumor agents, such as TRAIL, interleukins (IL-12, IL-4, and IL-15), immune checkpoint inhibitors (anti-PD-1 antibody), immune-enhancing stimulators (OX40L and GM-CSF), tumor suppressors (PTEN and P53), E-cad and Flt3L, and are systemically administered to GBM sites. Afterward, OVs bind to certain receptors to infect and enter tumor cells, resulting in tumor lysis. OVs that are armed with immune factors can enhance antiglioma efficacy by recruiting immune cells, which include T cells, NK cells, and macrophages, to the GBM sites. These activated immune cells can secrete certain antitumor cytokines, including IFN-γ, IFN-β, TNF-α, TNF-β, IL-2, and IL-6, which ultimately induce tumor cell apoptosis by specific signaling pathways. In summary, OVs expressing immunomodulatory transgenes effectively lead to tumor recession by the combination of virotherapy and immunotherapy.

This review provides a summary of ongoing studies on engineered oncolytic viruses that express immunomodulatory transgenes for the treatment of malignant gliomas, as shown in Table 1. The therapeutic patterns of oncolytic viruses with immunomodulatory transgenes for glioblastoma treatment are particularly illuminated in Fig. 1. The oncolytic virus can be genetically engineered to express immune cytokines and to augment immune responses by enhancing immune cell infiltration and stimulating subsequent cascade immune networks. As such, these modified oncolytic viruses can be exploited and serve therapeutic advantages against gliomas.

**Table 1:** A summary of currently open studies that utilize oncolytic viruses that express immunomodulatory transgenes against malignant gliomas.

Immunomodulatory transgenes	Virus types	Delivery, Combination	Genetic alteration	Refs
IL-15	ADV	Intratumoral, CTL cells	E2F-1 promoter replaced E1A promoter	^27^
IL-12	HSV	Intratumoral, anti-CTLA-4/PD-1	Deletions of γ34.5 and α47, LacZ insertion	^29^
IL-4	HSV-1 HSV-1	Intratumoral, single agent Intratumoral, single agent	Deletion of γ_1_34.5, α27-tk insertion Deletion of γ_1_34.5, α27-tk insertion	^30,33^
TRAIL	ADV	Subcutaneous, single agent	H5CmTERT promoter, Rb mutation	^36^
Anti-PD-1	HSV-1	Intratumoral, single agent	Deletions of γ34.5, LacZ insertion	^57^
OX40L GM-CSF PTEN	ADV VV ADV	Intratumoral, anti-PD-L1 Subcutaneous, rapamycin Intratumoral, single agent	Deletion of E1A gene, RGD-4C insertion LacZ insertion Deletions of El, E3 regions, and IX gene	^59,61,64^
P53	ADV	Intratumoral, single agent	E1 deletion	^66^
E-cad Flt3L	HSV HSV-1	Intratumoral, single agent Intratumoral, single agent	CDH1 gene insertion Deletions of γ_1_34.5, LacZ insertion	^69,73^

### Oncolytic viruses that express IL-12

IL-12 is a heterodimeric protein that plays a pivotal role in linking the innate and adaptive immune systems. Previous data have demonstrated that IL-12 is a cytokine with potent antitumor properties. It is produced by antigen-presenting cells, including B lymphocytes, dendritic cells, and monocytes. It serves to augment the cytolytic activities of NK cells and cytotoxic T lymphocytes and enables the development of a TH-1-type immune response^30,37,38^. In addition, the antiangiogenic properties of IL-12 have been identified and characterized^39–41^, which may represent a second potential mechanism for its antitumor activity. In recent years, researchers found that oncolytic viruses that express IL-12 could powerfully produce an antiglioma immune response in a glioma-bearing model^29,31^. Thus, the potential mechanisms of IL-12-mediated antitumor activity depend not only on the activation of the innate and adaptive effector immune systems but also on the inhibition of angiogenesis.

Based on the antitumor activity and antiangiogenic properties of IL-12, genes that express IL-12 have been inserted into the engineered oncolytic virus genome. In 2013, many studies reported that a genetically engineered oncolytic herpes simplex virus, when armed with the immunomodulatory cytokine interleukin 12, significantly enhanced the survival of tumor-bearing mice and effectively inhibited tumor growth^42–46^. Moreover, the safety and efficacy of the oncolytic virus were evaluated, and some strides were made in a clinical study. In 2017, Saha et al. found that the combination of an oncolytic HSV that expresses IL-12 with immune checkpoint inhibitors, including anti-CTLA-4 and anti-PD-1 antibodies, could potently eradicate glioma cells and extend the survival of glioma-bearing mouse models^29^. This combination provides new insight into glioma therapy. In brief, previous data and preclinical safety studies of another oncolytic HSV that expresses IL-12 also provide a compelling rationale for the clinical translation of IL-12-armed oncolytic HSVs for the treatment of patients with malignant gliomas.

### Oncolytic viruses that express TRAIL

One novel strategy for tumor treatment is to induce apoptosis of tumor cells. Tumor necrosis factor (TNF)-related apoptosis-inducing ligand (TRAIL) is a member of the tumor necrosis factor (TNF) superfamily and can induce apoptosis of tumor cells through activation of the TNF/CD95L axis and spare the majority of nonmalignant cells^47^. TRAIL is a strong therapeutic candidate for the treatment of glioblastoma because TRAIL can potently induce tumor-specific apoptosis^48^. With a deeper understanding of glioma therapy, TRAIL is widely used to exploit new therapeutic strategies. Most recent data have shown more potent antitumor efficacy of oncolytic viruses that encode TRAIL than of oncolytic viruses without TRAIL expression in xenograft models of subcutaneous and orthotopic glioblastoma, specifically through superior induction of apoptosis and the promotion of extensive viral distribution in tumor tissues^35,36,49^. Based on the properties of TRAIL-induced apoptosis of tumor cells, oncolytic viruses that express TRAIL could provide interesting approaches for glioma treatment.

### Oncolytic viruses that express IL-15

IL-15, which is a part of the 4a-helix bundle cytokine family, is a crucial factor in immune regulation and primarily comes into play through the activation of NK cells. It is a receptor complex that consists of three subunits: a unique a-chain, a b-chain (shared with IL-2), and a common g-chain. As a novel molecular agent, IL-15 is used in tumor research and shows powerful antitumor immune responses through the stimulation of natural killer cells^50–52^. In 2017, Rivera et al. found that IL-15 could decrease the migration, invasion, and proliferation of tumor cells and inhibit angiogenesis^53^. Moreover, oncolytic viruses that express IL-15 significantly lyse tumor cells and reduce the tumor volume by activating natural killer (NK) cells, CD8^+^ T cells, and other immune cells^27,28,54^. Furthermore, our research team found that the destruction capacity of oncolytic adenovirus armed with IL-15 on tumor cells was stronger than that of the control virus (data not shown). Thus, the above evidence indicates that oncolytic virus-encoded IL-15 generates antitumor efficacy by activating innate and adaptive effector immune mechanisms, as well as by inhibiting angiogenic activity. This opens a new therapeutic direction for patients with malignant gliomas. However, due to limited research on oncolytic viruses encoding IL-15 for glioma treatment, further studies are necessary.

### Oncolytic viruses that express immune checkpoint inhibitors

Immune checkpoints, especially PD-1, PD-L1, and CTLA-4, are immunosuppressive molecules that are upregulated in the GBM microenvironment^29^. Immune checkpoint blockers can regulate different inhibitory pathways on immune cells, overcome the immunosuppressive microenvironment, and further promote antitumor immunity through distinct and nonredundant immune evasion mechanisms^55^. Previous studies have demonstrated therapeutic efficacy with immune checkpoint inhibitors in orthotopic glioma-bearing models^29^. Moreover, Lukas et al. demonstrated the safety and efficacy of an immune checkpoint blocker (anti-PD-L1 antibody) in patients with recurrent GBM in a clinical trial^56^. In recent research, Passaro et al. confirmed that oHSV-1 encoding an anti-PD-1 antibody showed potent and durable antitumor immune responses after intratumoral administration and improved survival in preclinical GBM models^57^. Thus, OVs armed with immune checkpoint inhibitors may be a promising strategy for GBM patients in the future.

### Oncolytic viruses that express immune stimulators

OX40 ligand (OX40L) is an immune costimulator that binds the unique costimulator OX40 on T cells^58^, which makes it a good option for OV therapy to increase activation of T cells, which further recognize antigens on tumor cells infected with the virus. Its mechanism is due to tumor-specific immunity, and this modality is more tumor-specific than immune checkpoint blockade. Previously, an oncolytic adenovirus expressing the immune costimulator OX40L exhibited superior tumor-specific activation of lymphocytes and proliferation of CD8^+^ T cells specific to tumor-associated antigens, resulting in cancer-specific immunity^59^. In addition, granulocyte macrophage colony-stimulating factor (GM-CSF) is also a potent inducer of tumor-specific, long-lasting antitumor immunity in both animal models and human clinical trials^9^. Prior work demonstrated that oncolytic viruses expressing GM-CSF produced stronger antitumor immune responses in human solid tumors than oncolytic viruses not expressing GM-CSF^60^. Recent studies have investigated the efficacy and safety of oncolytic viruses encoding GM-CSF and their potentially enhanced immune activity in glioma-bearing models^61,62^. This tumor-specific strategy will be beneficial and efficacious for glioma patients in the future.

### Oncolytic viruses that express tumor suppressors

Phosphatase and tensin homolog (PTEN) is recognized as a candidate tumor suppressor gene based on the presence of inactivating mutations in several tumors^63^. Given its deletion and mutation in tumors, some studies have transfected the PTEN gene into glioma cells, and the expression of exogenous PTEN impeded the growth of glioma via augmentation of the immune response. Previous research revealed that recombinant adenovirus armed with the PTEN gene was able to inhibit the proliferation and tumorigenicity of glioma cells^64^. In addition, P53 is also a tumor suppressor gene involved in several aspects of cell cycle control and suppression of transformation. P53-armed virus significantly decreased tumor volume and prolonged the survival of mice compared with control virus in GBM models^65,66^. A recombinant adenovirus expressing p53 in glioma cells led to biological effects of the newly expressed p53 protein, which induced apoptosis to produce rapid and generalized death of human glioma cells^67^. Therefore, using recombinant viruses for the delivery of tumor suppressor genes into glioma cells is a promising, rational, and effective approach to treat glioma based on the transfer of genes.

### Oncolytic viruses that express E-cadherin

E-cadherin (E-cad) is a calcium-dependent cell–cell adhesion molecule that cooperates with nectin-1 in the formation of cell–cell adherent junctions. E-cad binding to the receptor KLRG1 protects E-cad-expressing cells from being lysed by NK cells. Overexpression of E-cad on virus-infected cells may directly facilitate cell-to-cell infection, which is the dominant method of intratumoral viral spread^68^. Recent studies have shown that oncolytic herpes viruses expressing E-cadherin inhibit the clearance of NK cells to enhance viral spread and cause tumor regression, and oHSVs encoding E-cad remarkably prolongs the survival rate in GBM-bearing mouse models^69^. This suggests that E-cad can be added as a module to oncolytic viruses to improve therapeutic efficacy in GBM.

### Oncolytic viruses that express Flt3L

FMS-like tyrosine kinase 3 ligand (Flt3L) stimulates maturation and proliferation of DCs and NK cells. Flt3L protein could retard tumor progression and decrease the number of tumor metastases^70^. In 2006, Curtin et al. investigated an adenoviral vector expressing Flt3L (AdFlt3L) that induced a specific increase in the levels of IFN-α secreted by DCs in the brain^71^. Moreover, AdFlt3L prominently suppressed glioma growth and improved survival when tumors were treated within 3 days of implantation in an intracranial glioma model by creating an inflammatory environment^72^. In addition, Barnard et al. found that oHSVs expressing Flt3L significantly extended life expectancy in glioma-bearing mice^73^. This is a novel approach in which virus-mediated expression of Flt3L leads to either eradication of the tumor or significant extension of the life span of animals.

## Potential pitfalls

Although oncolytic viruses that are armed with immunomodulatory transgenes for malignant glioma treatment have become increasingly popular, all oncolytic virus-based therapies, like other therapies, appear to be limited in their effectiveness. For example, the host responds to the oncolytic virus by inducing intratumoral infiltration of macrophages that can engulf the virus, limiting the potential of this therapeutic strategy. Some previous studies on the therapeutic effects of oncolytic viruses encoding immunomodulatory transgenes for malignant glioma treatment revealed potential pitfalls.

### Tumor microenvironment

**Fig. 2:**
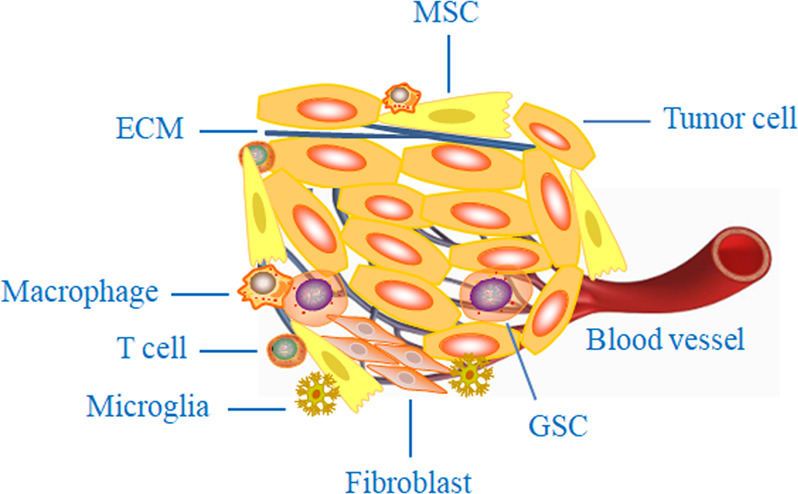
Diagram of the tumor microenvironment. The composition of the TME is extremely complex, mainly consisting of tumor cells, fibroblasts, endothelial cells, GSCs, mesenchymal stem cells (MSCs), TAMs, the extracellular matrix (ECM), and microglia, as well as cytokines and chemokines. The TME is recognized as an important determinant of tumor progression and therapeutic resistance.

The tumor microenvironment (TME) is increasingly recognized as an important determinant of tumor progression and therapeutic resistance. A thorough understanding of the properties and functions of the TME is required to obtain a complete understanding of brain tumor biology and treatment. Generally, the tumor microenvironment consists of tumor cells, fibroblasts, endothelial cells, tumor stem cells, mesenchymal stem cells (MSCs), tumor-associated macrophages (TAMs), the extracellular matrix, and microglia, as well as cytokines and chemokines that are secreted by tumor and stromal cells^74–78^, as shown in Fig. 2. Previous work has shown that the TME can be improved and remodeled by external agents and factors, including spleen tyrosine kinase, salidroside, microvesicles, exosomes, and irradiation^79–83^. Over many decades, research groups have shown that oncolytic viruses can modulate changes in the TME and further generate antitumor immunity^84–87^. However, the regulatory mechanism of oncolytic viruses remains unclear, and adverse consequences of TME alteration have been gradually explored and reported.

**Fig. 3:**
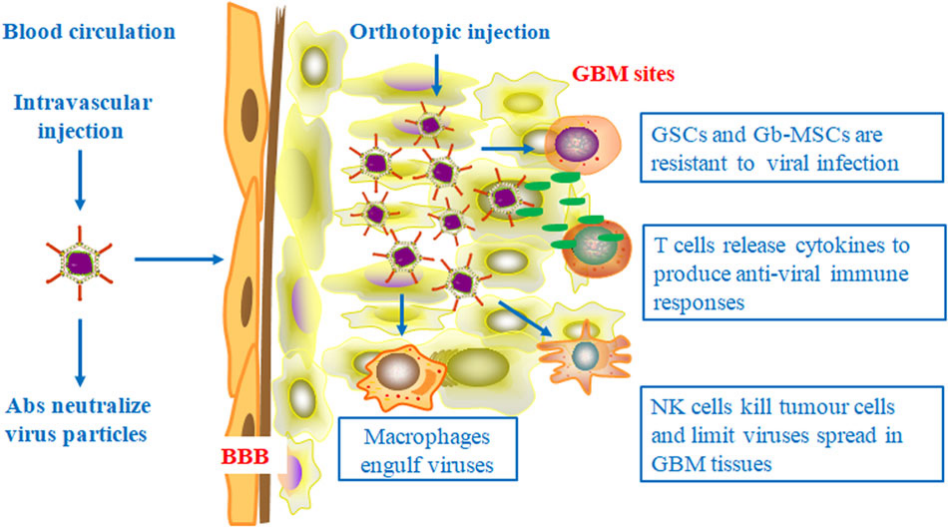
Schematic summarizing the events and factors that are related to the limited effectiveness and spread of oncolytic viruses in GBM treatment. Oncolytic viruses that express immunomodulatory transgenes are administered to the host by systemic vascular delivery or orthotopic injection. Systemic injection of OVs can be neutralized by Abs and blocked by the blood–brain barrier, and the OVs administered by orthotopic injection are engulfed and destroyed by activated immune cells, limiting viral spread in GBM tissues. In addition, GSCs and Gb-MSCs in the tumor microenvironment may be resistant to viral infection, which decreases therapeutic efficacy.

It is also important to seek understanding of how oncolytic viruses are affected by the TME in terms of differences between the TME and the natural milieu of the viruses. With a further understanding of the TME, researchers have demonstrated that immune infiltrating cells in the GBM microenvironment not only attack exogenous viruses but also restrict their spread to tumor cells and surrounding sites^88–92^, which may be a pitfall of immunomodulatory therapy, as shown in Fig. 3. TAMs are one of the major components in the TME. Although the shift from the protumor M2 (TAM2) to the antitumor M1 (TAM1) phenotype can obviously augment antiglioma effects^93,94^, macrophages can engulf and eliminate virus particles, which results in a significant reduction in virus titer in tumor sites. Recent studies have demonstrated that TAMs and microglia can limit the replication and spread of oncolytic viruses^88,89^. Myeloid-derived suppressor cells (MDSCs) are known to act as major immunosuppressive cells within the GBM microenvironment. MDSCs induce B cell-mediated immunosuppression, perhaps inhibiting the effective response to oncolytic viruses armed with immunomodulatory transgenes^95^. In addition, the blood–brain barrier (BBB) limits the delivery of viral vectors, greatly compromising their oncolytic efficacy^25^. Moreover, related investigations have shown that GSCs and glioma-associated mesenchymal stem cells (Gb-MSCs) in the TME are likely to generate resistance to oncolytic virus therapy^78,87,96^. Therefore, the number of effective viruses that arrive at the targeted sites will be greatly reduced. In 2017, Pulluri et al. reported that TME changes could generate resistance to immune checkpoint inhibitors in metastatic melanoma^97^, which indicates the possibility of cytotoxic properties in the glioma microenvironment. Last, these data raise controversial issues regarding the association between the TME and oncolytic viruses in terms of benefits or harm for efficacy. Thus far, there has not been a conclusive finding^98^. The previously mentioned pitfalls must be seriously considered, and potential mechanisms need to be further exploited. These data strongly suggest that the tumor microenvironment does pose potential safety hazards in terms of oncolytic virotherapy for malignant gliomas.

### Do oncolytic viruses that express immunomodulatory transgenes support or suppress tumor angiogenesis?

Angiogenesis, the process by which new blood vessels develop from the preexisting vascular endothelium, plays a crucial role in solid tumor recurrence and progression. Hence, antitumor vascular therapy has also become a popular approach for malignant gliomas. However, it is worth mentioning that there is no definite conclusion on whether oncolytic viruses that express immunomodulatory transgenes support or suppress tumor angiogenesis. These confusing issues are interesting for researchers and the basis of several promising studies. In 2012, Sahin et al. found that oncolytic viral therapy with the oncolytic virus HF10 contributed to tumor angiogenesis and thus supported tumor growth; further, this may possibly have been due to the inflammatory response that was induced by viral infection and viral proteins that were expressed during viral replication^99^. The mechanisms that underlie enhanced angiogenesis are not precisely known, but preclinical data regarding herpetic stromal keratitis in wild-type HSV-1 infection revealed that angiogenesis may be induced by paracrine effects that result from the release of VP22 or CpG motifs in the HSV-1 DNA, which are required for viral replication^100,101^. However, previous studies demonstrated that oncolytic viruses could disrupt tumor vascular endothelial cells and inhibit angiogenesis by directly infecting and killing vascular endothelial cells^102–104^. Moreover, our research team found that the tube segment lengths of HUVECs treated with oncolytic adenovirus were significantly shorter than those of the controls. The potential mechanism may involve the oncolytic virus inhibiting tumor angiogenesis by targeting VEGF^104^. In addition, researchers have also reported that TRAIL significantly enhanced the angiogenic activity and migration ability of human microvascular endothelial cells in vitro and in vivo^105,106^. Additional data suggest that TRAIL primarily inhibits angiogenesis by inducing vascular endothelial apoptosis, which leads to vessel recession^107–110^. Moreover, IL-15 and IL-12 could significantly decrease the number of blood vessels, which generates antiangiogenic effects^39–41,53^. Nevertheless, there is apparently no definitive conclusion for whether oncolytic viruses that express immunomodulatory transgenes support or suppress tumor angiogenesis.

To avoid further confusion, these pitfalls must be thoroughly studied and resolved. With potential breakthroughs, a new era will begin for patients with malignant gliomas. Oncolytic virus-encoded immune cytokines have shown different abilities to promote or suppress tumor angiogenesis under different conditions. If these oncolytic viruses expressing immunomodulatory transgenes were investigated as glioma treatments, the effects of their support or suppression of the angiogenic potential of gliomas should also be considered.

### The immune system combats exogenous viruses

Genetically engineered oncolytic viruses that are injected into the host can stimulate the immune system, generating a dual curative effect, which includes virus treatment and gene therapy. Research has shown that immune regulatory factors, including IL-4, IL-15, and IL-12, can enhance the activity of macrophages and neutrophils, contribute to the production and activation of natural killer cells and CD8^+^ T cells, and regulate cytokine production and memory T-cell survival and proliferation^29,33,54^. Thus, these complex elements could largely induce the apoptosis and lysis of tumor cells. Despite generating antitumor effects, the immune system also attacks the virus, which results in limited potency and expansion. This may explain why viruses cannot reach and kill distant tumors effectively.

Previous studies revealed that intravascularly injected viruses could be neutralized and eliminated by antibodies in the host^111–113^, as shown in Fig. 3. To overcome these hurdles, researchers have used careful orthotopic injection for malignant gliomas. However, activated macrophages and microglia engulf virus particles and limit their spread^25,88,89^. In 2015, Kober et al. found that microglia and astrocytes that were recruited in the TME preferentially cleared viral particles by immediate uptake after delivery, thus not allowing efficient viral infection^114^. As such, the efficacy and safety of genetically engineered oncolytic viruses must be guaranteed before wide application in clinical practice.

### Tumor heterogeneity

Tumor heterogeneity is one of the most common characteristics of malignant tumors that contributes to tumor progression and recurrence^115^. Data from multiple studies demonstrate that higher levels of intratumoral heterogeneity predispose patients to inferior responses to anticancer therapies^116^. Heterogeneity provides a novel understanding of therapeutic resistance; thus, an accurate assessment of tumor heterogeneity is necessary to develop effective therapies.

It is well known that intertumoral and intratumoral heterogeneity persist in GBM, which is complicated due to the diverse tumor cell origins that are shown in glioma-bearing models and clinical tumor specimens^117^. Previous studies using single-cell analysis have identified the great heterogeneity in different GBM subtypes and emphasized the clinical significance of GBM heterogeneity^118,119^. The TME and location of the tumor can influence intratumoral heterogeneity, while different molecular subtypes, such as the proneural and classical mesenchymal subtypes, influence intertumoral heterogeneity. In 2014, Reardon et al. revealed that tumor heterogeneity significantly influenced the outcomes of patients with glioblastoma^115^. Tumor heterogeneity makes tumors highly resistant to different therapeutic strategies, which results in decreased efficacy^116,120–122^. One possible mechanism of resistance involves the heterogeneous expression of the epidermal growth factor receptor (EGFR) in GBM^123^. This poses a substantial challenge for the effective use of EGFR-targeted therapies. In addition, our research team has repeatedly found that the sensitivity of different glioma cell lines to genetically engineered oncolytic adenoviruses is discrepant in research (data not shown), which suggests powerful treatment challenges for patients with malignant gliomas. Therefore, designing targeted therapies based on a range of molecular profiles can be a more effective strategy for eradicating treatment resistance, recurrence, and metastasis. Despite harvesting remarkable results in clinical trials, the therapeutic effects of oncolytic viruses on different types of malignant gliomas may be incredibly diverse. These data provide researchers with different insights into clinical treatment. Thus, a better understanding of glioblastoma heterogeneity is crucial for promoting therapeutic efficacy.

## Future perspectives

Oncolytic virotherapy is a promising approach in which viruses are genetically modified to selectively replicate in tumor cells. Furthermore, the efficiency and safety of these viruses have been demonstrated in preclinical and clinical studies. Due to the previously mentioned obstacles, the spread and concentration of oncolytic viruses in the TME are restricted. To overcome such obstacles, a better understanding of the TME and better designed strategies for improved therapeutic efficacy should be pursued in research on oncolytic viruses for malignant glioma treatment. Oncolytic viruses encoding immunomodulatory transgene therapy for malignant gliomas may become a popular approach that is widely used for clinical practice in the future. Furthermore, the immune checkpoint molecule known as PD-1 has become a research hot spot in tumor research. For cancers with an immunologically ‘cold’ TME (e.g., GBM), immune checkpoint blockade immunotherapy alone has not yet been successful^124–127^. However, one related study verified that the effect of oncolytic virus immunotherapy combined with anti-PD-1 antibody in glioblastoma was significantly better than that of oncolytic virus immunotherapy alone^29,125^. Thus, this combination should be translated to the clinic and tested against other immunosuppressive cancers.

The current approach for oncolytic virus administration mainly includes direct intratumoral injections and systemic vascular delivery. Stereotactic injections or resection bed inoculation are the most commonly studied and simplest methods for introducing viral vectors into high-grade gliomas. This approach has the advantage of bypassing the BBB and introduces a high concentration of viruses directly into the tumor tissues. However, it is limited by multiple factors in the host and only delivers a single dose. Currently, stem cell-based therapy for gliomas has emerged as a promising novel strategy. Given their inherent tumor-tropic migratory properties, stem cells can serve as vehicles for the delivery of therapeutic efficacy^128^. In 2008, Sonabend et al. reported that administration in MSCs enabled oncolytic virus delivery to distant glioma cells and that this delivery significantly enhanced the infection and apoptosis of tumor cells compared to injection alone, revealing a therapeutic advantage^129^. Moreover, in prior investigations, MSCs carrying oncolytic viruses were injected into the carotid artery of mice, and these cells migrated to tumor sites, which resulted in inhibited glioma growth and improved survival of glioma-bearing animals^130–132^. Since then, many studies using neural stem cells loaded with oncolytic adenoviruses have demonstrated extended delivery of the oncolytic virus and prolonged survival of glioma-bearing animals that were treated with the stem cell-mediated oncolytic virotherapy^133–135^. The given data reveal that stem cell vectors could improve the outcomes of oncolytic virotherapy for glioma treatment. With ongoing exploration of trans-differentiation techniques, the barrier of sourcing could be overcome. The advantages of combining stem cell vehicles with oncolytic virus-encoding immunomodulatory transgene therapy for patients with malignant gliomas may be a greater area of focus in the next few decades.

## Conclusions

Genetically engineered oncolytic virus therapy for malignant gliomas has recently been an increasing focus of research. Oncolytic viruses that express immunomodulatory transgenes generate antitumor effects via oncolytic and immunotherapy effects. Despite the numerous oncolytic virus-related studies that have produced great strides in the field of glioma treatment, serious obstacles remain. Moreover, side effects that are caused by virotherapy in clinical trials exist. It remains uncertain whether oncolytic viruses, once appropriately modified, can be used to treat different types of gliomas. These issues need to be further explored and explained in future studies. In conclusion, using oncolytic viruses that are armed with immunomodulatory transgenes for malignant glioma treatment is still in its early stages, and a better understanding of the biological consequences of this therapy is required before its wide use in treating patients with malignant gliomas.
