# Correction: Advances and potential pitfalls of oncolytic viruses expressing immunomodulatory transgene therapy for malignant gliomas

**DOI:** 10.1038/s41419-020-03216-z

**Published:** 2020-11-23

**Authors:** Qing Zhang, Fusheng Liu

**Affiliations:** 1 Brain Tumor Research Center, Beijing Neurosurgical Institute, Capital Medical University, Beijing, 100070 China; 2 Department of Neurosurgery, Beijing Tiantan Hospital Affiliated to Capital Medical University, Beijing, 100070 China; 3 Beijing Laboratory of Biomedical Materials, Beijing, 100070 China

Correction to: *Cell Death and Disease*

10.1038/s41419-020-2696-5 published online 25 June 2020

Since publication the authors have noticed that there was an error in the author information. Fusheng Liu is the only corresponding author, while Qing Zhang is the first author.

This has been corrected in the PDF and HTML versions.

